# Ifitm3 Limits the Severity of Acute Influenza in Mice

**DOI:** 10.1371/journal.ppat.1002909

**Published:** 2012-09-06

**Authors:** Charles C. Bailey, I-Chueh Huang, Christina Kam, Michael Farzan

**Affiliations:** Department of Microbiology and Immunobiology, Harvard Medical School, New England Primate Research Center, Southborough, Massachusetts, United States of America; Mount Sinai School of Medicine, United States of America

## Abstract

Interferon-induced transmembrane (IFITM) proteins are a family of viral restriction factors that inhibit the entry processes of several pathogenic viruses, including influenza A virus (IAV), *in vitro*. Here we report that IAV-infected knockout mice lacking the *Ifitm* locus on chromosome 7 exhibited accelerated disease progression, greater mortality, and higher pulmonary and systemic viral burdens as compared to wild type controls. We further observed that the phenotype of *Ifitm3*-specific knockout mice was indistinguishable from that of mice lacking the entire *Ifitm* locus. Ifitm3 was expressed by IAV target cells including alveolar type II pneumocytes and tracheal/bronchial respiratory epithelial cells. Robust Ifitm3 expression was also observed in several tissues in the absence of infection. Among murine *Ifitm* promoters, only that of *Ifitm3* could be induced by type I and II interferons. Ifitm3 could also be upregulated by the gp130 cytokines IL-6 and oncostatin M on cells expressing appropriate receptors, suggesting that multiple cytokine signals could contribute to Ifitm3 expression in a cell or tissue-specific manner. Collectively, these findings establish a central role for Ifitm3 in limiting acute influenza *in vivo*, and provide further insight into *Ifitm3* expression and regulation.

## Author Summary

The human genome contains many genes devoted to combating viral infections. Some of these genes encode a family of proteins called interferon-induced transmembrane (IFITM) proteins which were recently discovered to inhibit infection by influenza A viruses in cell culture experiments. Here we show that genetically engineered mice lacking the murine equivalents of the human *IFITM* genes are more susceptible to influenza than mice with a full complement of these genes. In addition, deletion of one of these genes alone, *Ifitm3*, made mice equally susceptible to infection, showing that the Ifitm3 protein plays a central role in the control of influenza A virus in living animals. We also show that murine Ifitm proteins are expressed on cells targeted by influenza A viruses and that the control of their expression in animals is more complex than suggested by previous cell culture studies.

## Introduction

The interferon-induced transmembrane (IFITM) proteins are a family of small transmembrane proteins that mediate some of the antiviral activities of type I and II interferons [1], [2], [3]. IFITM-mediated restriction is specific to particular virus families. Influenza A viruses (IAV), Ebola virus (EBOV), Marburg virus (MARV), SARS coronavirus (SARS-CoV), dengue virus, and West Nile virus are all efficiently restricted by one or more IFITM family proteins. Conversely, IFITM proteins do not restrict murine leukemia virus (MLV), a range of alphaviruses, and arenaviruses including Machupo virus (MACV), Lassa virus (LASV), and lymphocytic choriomeningitis virus (LCMV) [2], [4], [5]. Vesicular stomatitis virus is inefficiently restricted [6], [7]. Two groups have reported cell line-specific restriction of HIV-1 [1], [8], but these reports differ as to which cell lines show efficient restriction. Restriction activity against non-enveloped viruses has not been reported to date.

The mechanism of action of IFITM-mediated restriction has not yet been determined. Multiple lines of evidence indicate that restriction occurs after virion endocytosis but during or prior to membrane fusion. IFITM proteins prevent infection by retroviruses pseudotyped with entry proteins of IFITM-restricted viruses, but not those pseudotyped with entry proteins of unrestricted viruses [4]. Entry assays using virions containing β-lactamase-Vpr (BlaM-Vpr) fusion proteins likewise reveal that IFITM proteins prevent transfer of BlaM-Vpr to the cytosol of target cells [8]. Previously, we showed by fluorescent microscopy that labeled IAV virions are internalized normally and traffic to acidified endocytic compartments despite IFITM expression [4], a conclusion confirmed and extended by Feely et al. using a rigorous set of imaging studies. They further demonstrated by fluorescent *in situ* hybridization that IFITM proteins cause sequestration and accumulation of IAV genetic material in late endocytic/lysosomal compartments [9].

IFITM proteins also localize to late endosomes/lysosomes as demonstrated by co-localization with LAMP1, LAMP2, and CD63 [4]. Moreover, IFITM protein over-expression – and in some cell lines, interferon treatment – results in enlargement of these organelles [9]. Finally, a recent report showed that endosomes in interferon-treated *Ifitm* knockout cells are inefficiently acidified and suggested that a physical association between Ifitm3 and a subunit of the vacuolar ATPase complex mediates this effect [10]. It appears, therefore, that IFITM proteins alter the properties of late endosomes and lysosomes, and render these organelles inhospitable to viral fusion. Such a model is supported by the pattern of IFITM-mediated restriction; excepting HIV-1, restricted viruses generally fuse late in the endocytic pathway whereas unrestricted viruses fuse at the plasma membrane or in early endocytic compartments. Further evidence that restriction depends on the site of viral fusion comes from investigation of SARS-CoV. SARS-CoV requires lysosomal cathepsins to activate its entry protein and fuse with the lysosomal membrane [11]. Treating receptor-bound virions with trypsin, however, removes this cathepsin dependency, induces fusion at the plasma membrane, and bypasses IFITM-mediated entry restriction [4].

Most vertebrates have two or more *IFITM* genes [12]. The human *IFITM* family is composed of four functional genes, *IFITM1*, *2*, *3*, and *5*. Murine *Ifitm1*, *2*, *3*, and *5*, located on chromosome 7, are clear orthologs of their human counterparts but mice possess two additional loci: *Ifitm6*, also located on chromosome 7, and *Ifitm7*, a retrogene on chromosome 16 [13]. The proteins expressed by these genes restrict influenza A virus (IAV), filovirus, flavivirus, and SARS coronavirus entry *in vitro* with varying efficiencies [4], [6]. *Ifitm5* expression is limited to bone [14], [15], and the roles of *Ifitm6* and *7* are not yet clear. Two strains of knockout mice were used in the following experiments. *IfitmDel* mice lack *Ifitm1*, *2*, *3*, *5*, and *6* and *Ifitm3egfp* mice lack *Ifitm3* alone [13].

The aim of the present study was to determine the *in vivo* contribution of the Ifitm proteins to the innate immune control of IAV. We show that Ifitm3 alone makes a significant contribution to the control of influenza in mice and provide further insight into its expression and regulation.

## Results/Discussion

### Murine Ifitm proteins are protective against influenza *in vivo*

**Figure 1:**
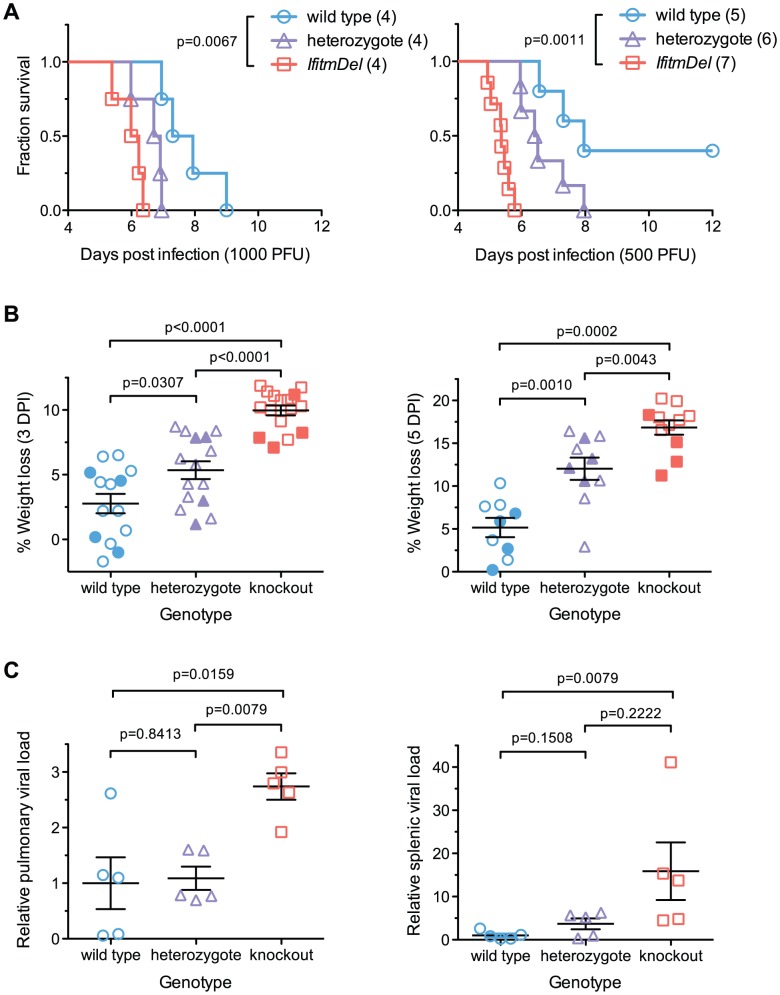
An *Ifitm* locus deletion renders mice more susceptible to influenza. *IfitmDel* knockout mice challenged intranasally with either 1000 or 500 PFU of influenza A/PR/8/34 (H1N1) (PR8) exhibited more rapid disease progression and higher mortality than wild type and heterozygous controls. (**A**) At 1000 PFU, a survival analysis between wild type and knockout animals showed significant differences (p = 0.0067, Mantel-Cox test) in survival time as did a comparison of wild type and heterozygous mice (p = 0.0266). With a 500 PFU challenge, survival differences between all pairs of groups were statistically significant: wild type and knockout (p = 0.0011), wild type and heterozygous (p = 0.0366), heterozygous and knockout (p = 0.0012). At both challenge doses, heterozygous animals exhibited an intermediate phenotype consistent with a gene dosage requirement for the antiviral effects of *Ifitm*. (**B**) Weight loss measurements from all surviving study animals at 3 and 5 DPI are shown. Closed symbols represent animals challenged with 1000 PFU and open symbols represent animals challenged with 500 PFU. At both time points, weight loss differences between any two genotypes were statistically significant (Mann Whitney U test). Bars show mean ± SEM. (**C**) Pulmonary and systemic viral loads were measured at 3 DPI from animals challenged with 500 PFU of PR8. The relative abundance of IAV M2 transcript levels from total lung or spleen RNA, normalized to endogenous *Gapdh* transcript, is shown. Values represent fold changes relative to the mean viral burden of wild type mice. Pulmonary viral loads were 2.7-fold higher in knockout than in wild type controls (p = 0.0159, Mann Whitney U test). Error bars show mean ± SEM. Splenic viral loads were 15.9-fold greater in knockout than wild type animals (p = 0.0079), but splenic viral loads of wild type animals were barely above the detection threshold of the assay. Splenic viral loads of heterozygotes were not significantly different from those of wild type or knockout animals but a non-significant trend was evident across all three genotypes.

To determine the *in vivo* relevance of Ifitm proteins, we challenged cohorts of *IfitmDel*, wild type, and heterozygous male mice with intranasal doses of either 500 or 1000 PFU of influenza A/PR/8/34 (H1N1) (PR8). In accordance with institutional policies, mice were considered moribund and euthanized upon loss of 20% of initial body weight, defined as the 2-day average of their pre-inoculation weights. At both challenge doses, knockout mice exhibited significantly accelerated disease progression and mortality compared to wild type controls (Figure 1A). Heterozygotes had an intermediate phenotype. A modest but insignificant difference was observed in the mean survival time of knockout mice receiving each challenge dose, with mice receiving 1000 PFU surviving 0.7 days longer. With the 500 PFU challenge dose, two of five wild type animals survived acute infection but disease remained uniformly lethal in knockout mice. Weight loss at days 3 and 5 was significantly increased in *IfitmDel* and heterozygous animals as compared to wild type controls (Figure 1B; plots of body weight in Figure S1A).

Weight loss in wild type animals receiving a 500 PFU challenge followed trends reported by other investigators [16]. Price et al. found that mice generally maintained body weight until day 4–5 post-infection, after which they underwent a rapid period of weight loss that continued until day 8–9. The rapid clinical progression beginning on day 4–5 coincided with the onset of cellular and humoral immunity while recovery on day 8–9 corresponded to clearance of the virus. In contrast to wild type animals, *IfitmDel* mice began a steep decline in weight beginning on day 2 consistent with a failure of innate control of early infection. The variability in the rate of disease progression of knockout mice was also far lower than that of both wild type and heterozygous animals (Figure S1A). These results demonstrate that Ifitm proteins mediate innate immunity to influenza A virus during acute infection and that the effect of their deletion is powerful enough to overwhelm other sources of experimental variation. The intermediate phenotype of heterozygous animals shows that gene dosage is important for Ifitm protein-mediated viral restriction. The requirement for high gene dosage may also explain the duplication of the *Ifitm* alleles observed in multiple species.

### Viral loads are higher in infected *IfitmDel* knockout mice

Viral loads were measured in a separate cohort of mice euthanized three days after infection, the period during which body weights of wild type and *IfitmDel* animals diverge (Figure S1A). Weight loss trends in this cohort of animals were similar to those of previous cohorts (Figure S1B). Viral loads, as assessed by real-time RT-PCR of lung RNA, were 2.7-fold higher on average (p = 0.0159) in *IfitmDel* mice as compared to wild type controls (Figure 1C). Differences in splenic viral loads (Figure 1C) were similarly increased (15.9-fold, p = 0.0079).

Although the relative difference in splenic viral loads was high, IAV genome copy numbers in the spleen were on the order of 10^4^ to 10^5^ times lower than those in the lung (Figure S1C) and genome expression in the spleens of wild type animals was barely above the detection threshold of our assay. Similarly, we did not identify splenic lesions or any other lesions beyond those in the lungs, trachea, and nasal passages, consistent with the dependence of PR8 hemagglutinin on respiratory proteases for cleavage and activation [17], [18], [19], [20]. We therefore speculate that increased splenic viral loads in knockout mice are a result of more robust infection in the lung and subsequent leakage of virus or viral debris into the periphery.

### *Ifitm3*-specific and *IfitmDel* knockout mice are equally susceptible to IAV infection

Previously, we reported that murine embryonic fibroblasts (MEFs) derived from *IfitmDel* knockout mice are highly susceptible to IAV and that both baseline and interferon-induced Ifitm protein expression contribute to the IAV resistance in wild type MEFs [2]. Ifitm3 expression alone was sufficient to restore IAV entry restriction to knockout fibroblasts [9]. We have shown that over-expression of most murine Ifitm proteins in a human cell line confers some level of IAV restriction but that Ifitm3 is most effective while Ifitm5, 6, and 7 posses comparatively little activity [4]. We confirmed this observation in the context of *IfitmDel* MEFs (Figure S2A); again murine Ifitm3 demonstrated the most potent IAV restriction activity regardless of HA type, likely in part due to its higher steady-state expression level (Figures S2B and S2C).

**Figure 2:**
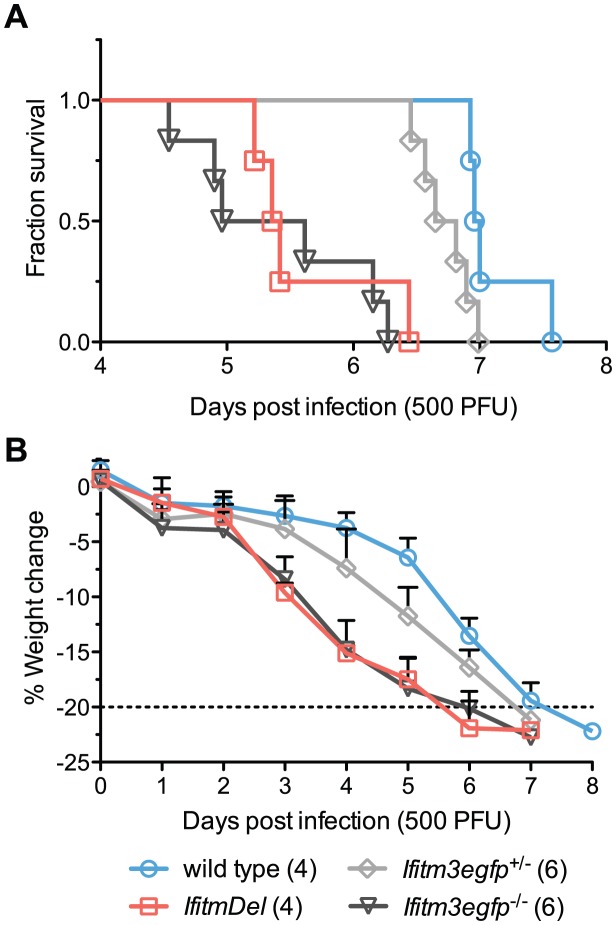
*IfitmDel* and *Ifitm3*-specific knockout mice exhibit indistinguishable phenotypes. *IfitmDel* knockout, *Ifitm3egfp^−/−^* (*Ifitm3*-specific knockout), *Ifitm3egfp^+/−^*, and wild type mice were challenged with 500 PFU of intranasal PR8. No differences were observed in disease progression between *IfitmDel* and *Ifitm3egfp* knockouts. *Ifitm3egfp* heterozygotes exhibit an intermediate phenotype similar to that of *IfitmDel* heterozygotes in Figure 1. (**A**) Survival times of *Ifitm3egfp* and *IfitmDel* knockouts were significantly different from those of wild type animals (p = 0.0038 and p = 0.0067 respectively, Mantel-Cox test) but not significantly different from one another (p = 0.5380). (**B**) Mean+SD weight loss over time is shown for mice in Figure 2A.

Although Ifitm3 most effectively restricts IAV *in vitro*, most human and murine Ifitm proteins restrict IAV to some extent when over-expressed [4]. To determine the specific *in vivo* contribution of Ifitm3, we performed an additional intranasal challenge of wild type, *IfitmDel*, and *Ifitm3*-specific (*Ifitm3egfp*) knockout mice with 500 PFU of PR8. Mortality and weight loss trends of *IfitmDel* and wild type animals were similar to those of Figure 1 above. Survival times of *Ifitm3*-specific knockout mice were not significantly different from those of *IfitmDel* knockout mice (p = 0.5380) and the weight loss curves for these two genotypes were superimposed (Figures 2A and B). As with the *IfitmDel* mice, heterozygotes of the *Ifitm3*-specific knockout mice exhibited an intermediate phenotype. We therefore conclude that, among Ifitm proteins, only Ifitm3 makes a substantial contribution to PR8 resistance *in vivo*.

### Ifitm3 expression is both cell-type and cytokine dependent

**Figure 3:**
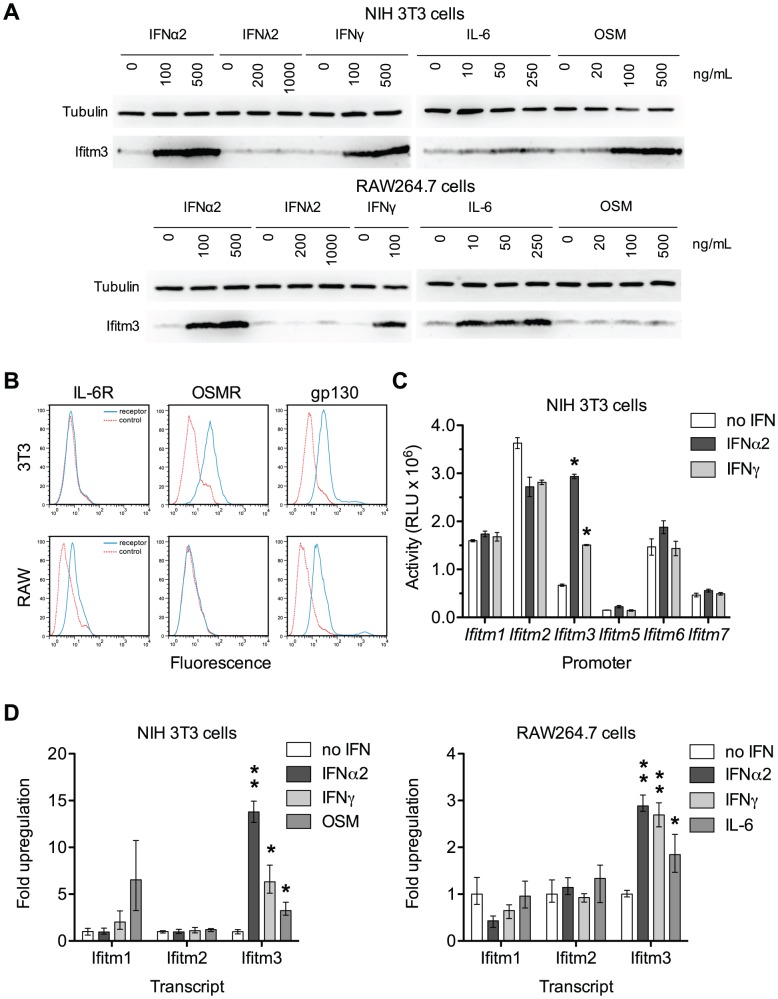
Both interferons and gp130-mediated cytokines induce Ifitm3 expression. Ifitm3 protein expression can also be induced by gp130-family cytokines in cells expressing appropriate receptors. The *Ifitm3* promoter alone among murine Ifitm promoters is interferon responsive. (**A**) Expression of Ifitm3 was determined by western blot in NIH 3T3 and RAW 264.7 cells treated for 2 days with the indicated cytokines. IFNλ2 failed to induce Ifitm3 expression at similar concentrations, likely due to lack of the type III IFN receptor. The gp130-family cytokine oncostatin M (OSM) was a potent inducer of Ifitm3 expression in 3T3 cells whereas RAW264.7 cells were responsive to IL-6, but not OSM. (**B**) Surface expression of the OSM receptor, IL-6 receptor, and gp130 by NIH 3T3 and RAW264.7 cells was determined by flow cytometry. Solid blue lines correspond to the fluorescence signal of the indicated receptor-binding antibodies and red lines show fluorescence of the isotype control. The responsiveness of 3T3 and RAW cells to OSM and IL-6 respectively (as shown in panel A) correlates with their expression of their corresponding receptors. (**C**) Activities of the six murine *Ifitm* promoters were assayed in mock-treated, IFNα2-treated, and IFNγ-treated NIH 3T3 cells transfected with plasmids encoding luciferase under the control of the indicated promoters. *Ifitm1*, *2*, and *6* promoters appear constitutively active, and in contrast to human orthologs, insensitive to interferon stimulation. The *Ifitm5* promoter shows almost no activity under any condition, consistent with its tissue-restricted expression *in vivo* [15]. The *Ifitm3* promoter alone is interferon responsive (4.4-fold induction with IFNα2; 2.2-fold with IFNγ). Bars show mean of triplicates ± SEM. Asterisk indicates p<0.0001 (two-factor ANOVA Bonferroni post test). (**D**) Cytokine regulation of *Ifitm1*, *2*, and *3* was confirmed at the mRNA level by qRT-PCR in both 3T3 and RAW cells. Bars depict fold upregulation of the indicated transcripts following a 24-hour incubation with the indicated cytokines relative to transcript levels in untreated cells. Ifitm3 transcript levels were significantly upregulated by IFNα2 (500 ng/ml) and IFNγ (100 ng/ml) in both cells lines. As in panel (A), OSM (250 ng/ml) upregulated Ifitm3 in 3T3 cells and IL-6 (250 ng/ml) upregulated Ifitm3 in RAW cells. Ifitm1 and Ifitm2 transcript levels were not increased by interferons. Mean Ifitm1 expression increased in response to OSM treatment but the difference was not significant due to the high variance of these samples. Error bars show mean ± range of triplicates. Asterisk indicates p<0.05 and double asterisk indicates p<0.01 (1-tail t-test assuming heteroscedastic samples).

Because the *Ifitm3* promoter is sensitive to interferon signaling we reasoned that other Jak/STAT-mediated cytokines might likewise induce Ifitm3 expression. We tested the ability of a type III interferon (IFNλ2), the acute phase cytokine IL-6, and oncostatin M (OSM), a cytokine released from activated dendritic cells that synergizes with type I interferon [21], to upregulate Ifitm3 expression. The latter two molecules belong to a diverse family of cytokines that signal through hetero- or multimeric receptors that activate the Jak/STAT pathway via a common gp130 subunit [22]. Consistent with previous studies, type I and type II interferons induced Ifitm3 expression in both NIH 3T3 murine fibroblasts, and the murine macrophage cell line, RAW264.7. IFNλ2 was ineffective, likely due to lack of the appropriate receptor. In 3T3 cells, however, oncostatin M (OSM) induced strong Ifitm3 expression and IL-6 was a potent inducer of Ifitm3 expression and in RAW264.7 cells (Figure 3A). The ability of these two cytokines to modulate Ifitm3 expression correlated with the surface expression of their cognate receptors (Figure 3B), and was not due to increased expression of type I or II interferons (Figure S3). These data raise the possibility that other gp130-mediated cytokines such as IL-11, IL-27, and LIF could modulate basal and inducible *Ifitm3* expression in cells bearing appropriate receptors. Furthermore, they indicate greater specificity and complexity in the regulation of *Ifitm3* expression, and possibly other interferon-stimulated genes, than previous studies have implied.

Human *IFITM1*, *2*, and *3* are interferon-stimulated genes [23]. Murine *Ifitm3* is shown to be interferon-inducible but the regulation of the other murine *Ifitm*s, particularly the mouse-specific family members *Ifitm6* and *7*, has not been studied. Therefore, we assayed the responsiveness of the murine *Ifitm* promoters to both type I and type II interferons (Figure 3C). We observed that, of the murine *Ifitm* genes, *Ifitm3* alone has an interferon-inducible promoter. Figure 3C also suggests that – at least under these tissue culture conditions – *Ifitm* promoters may be constitutively active, perhaps contributing to an intrinsic antiviral state. We validated the results of this promoter assay by analyzing cytokine-mediated upregulation of murine *Ifitm1*, *2*, and *3* transcripts by qRT-PCR. Consistent with the promoter studies, and in contrast to the behavior of their human orthologs, murine *Ifitm1* and *2* were unresponsive to both type I and type II interferons. *Ifitm3* transcript levels were significantly upregulated by both IFNα2 and IFNγ in both NIH 3T3 and RAW264.7 cells. *Ifitm3* was similarly induced by OSM in 3T3 cells and IL-6 in RAW cells consistent with both western blot and promoter data (Figure 3D). *Ifitm1* also appeared to respond to OSM stimulation although this increase was not statistically significant.

### Ifitm3 localization is consistent with its role as an IAV restriction factor

**Figure 4:**
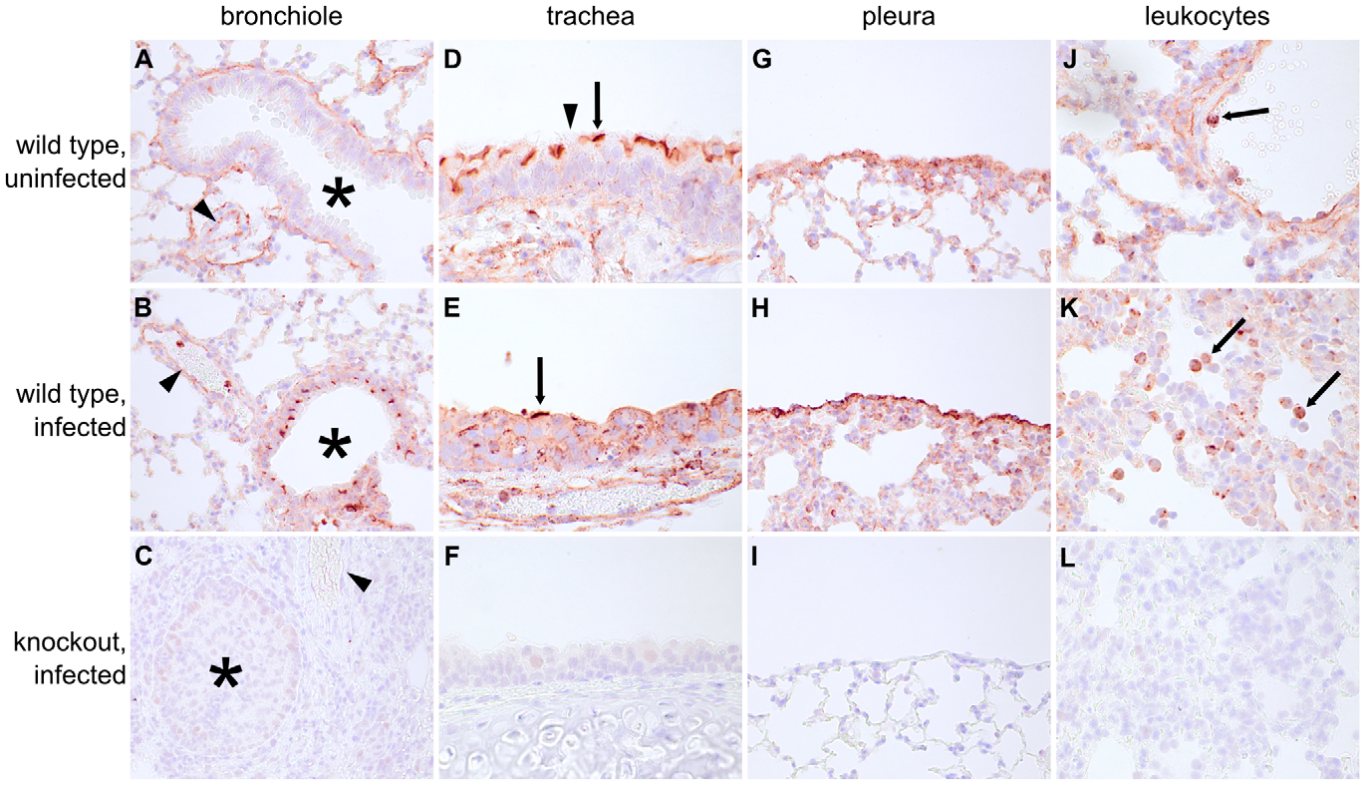
Ifitm3 is constitutively expressed by many respiratory tissues and induced in lower airway epithelium by influenza. Many lung tissues constitutively express Ifitm3. Panels show Ifitm3 expression (stained red/brown) in uninfected wild type (top) and infected wild type (6 DPI, middle) animals. Comparable tissues from knockout mice (bottom) are included as negative controls. (**A**–**C**) Ifitm3 expression was induced on lower airway (*) epithelium following influenza infection. Constitutive expression was observed on the endothelium of blood vessels (arrowheads). The endothelial cells in (A) and (B) provide a reference for staining intensity between the two slides. (**D**–**E**) In contrast to lower airway epithelial cells, upper airway epithelial cells constitutively express Ifitm3. Ifitm3 localizes to the apical borders of ciliated epithelial cells (arrows, inset shows magnified view of indicated cell in which the cilia can be visualized). In the infected animal in (E), the lesioned epithelium has become dysplastic. The flattened, non-ciliated epithelial cells show punctate cytoplasmic, rather than apical, staining. Interspersed goblet cells (arrowhead) do not stain. (**G**–**I**) Ifitm3 is constitutively and robustly expressed by the visceral pleura. (**J**–**L**) Leukocytes (arrows) often strongly express Ifitm3. The arrow in (J) indicates a circulating leukocyte. In (K), Ifitm3 expression can be seen on inflammatory cells recruited to a pneumonia lesion.

The data in Figures 2 and 3 show that Ifitm3 is the most potent IAV restriction factor of the murine Ifitm proteins and that Ifitm3 expression alone is efficiently induced interferons. To determine where and under what conditions Ifitm3 is expressed *in vivo*, we performed comprehensive immunohistochemical staining of the lungs of infected and uninfected wild type mice to determine baseline and influenza-induced patterns of Ifitm3 expression. Contrary to expectations, constitutive expression of Ifitm3 was seen in many lung tissues; marked induction was seen only in the respiratory epithelium of lower airways (Figure 4A, B, C). The pattern of distribution was similar in the remaining lung tissue in IAV-infected and uninfected mice. Constitutive expression was observed in respiratory epithelial cells of the upper airways (Figure 4D, E, F), the visceral pleura (Figure 4G, H, I), and in leukocytes (Figure 4J, K, L), suggesting that Ifitm3 may provide protection against influenza prior to the interferon and acute-phase responses. No immunostaining of *Ifitm3*-specific knockout mice was observed, confirming the specificity of the antibody for Ifitm3 (Figure S4).

**Figure 5:**
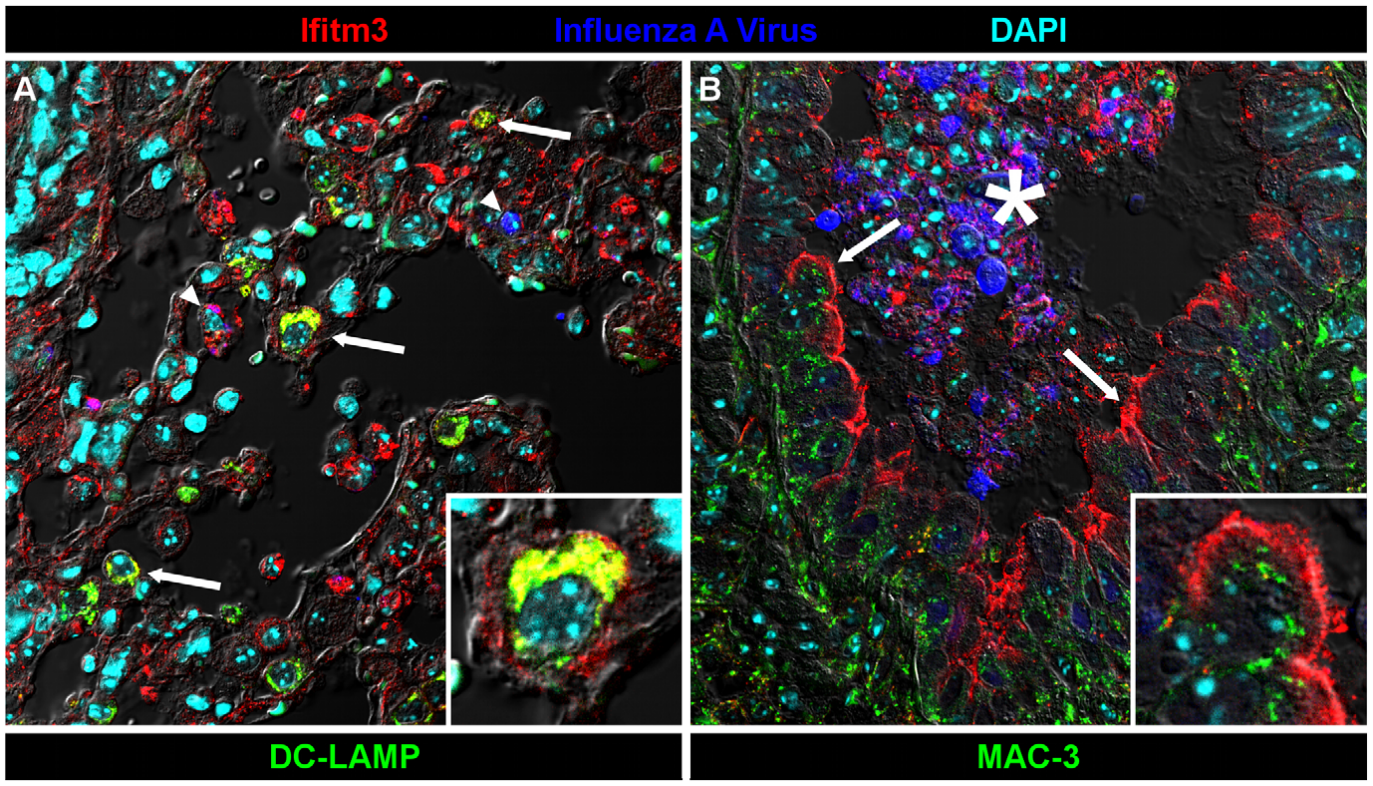
Ifitm3 is expressed by the target cells of influenza A virus. Ifitm3 is expressed by the target cells of IAV in infected wild type animals. Laser-scanning confocal micrographs show Ifitm3 (red), influenza A virus antigen (blue), nuclear chromatin (cyan), and either type II pneumocyte marker DC-LAMP (left, green) or lysosomal marker MAC-3 (right, green), against a differential interference contrast background. Tissues shown were harvested from an infected wild type animal at 6 DPI. (**A**) Ifitm3 is expressed on alveolar type II pneumocytes (arrows) marked by DC-LAMP (green), many of which are Ifitm3 positive (yellow). Occasional infected cells (arrowheads) including an infected Ifitm3 positive cell (magenta) are scattered throughout the parenchyma. Inset: Ifitm3 colocalizes with the lysosome-related organelle marker of type II pneumocytes, DC-LAMP (colocalization depicted in yellow). (**B**) Ifitm3 is localized to the apical membranes of ciliated respiratory epithelial cells (arrows) lining a bronchiole filled with IAV-infected (blue), necrotic, and Ifitm3-positive cellular debris (*). Ifitm3 does not colocalize with lysosomal marker MAC-3 (green) in these cells. Inset: magnified view of ciliated cell.

We also localized Ifitm3 expression specifically to influenza A virus target cells – alveolar type II pneumocytes and ciliated respiratory epithelial cells [24] – by immunofluorescence and confocal microscopy. Type II pneumocytes were identified by DC-LAMP expression, a marker of type II pneumocyte-specific lysosome-related organelles [25]. Ifitm3 is not only expressed by DC-LAMP positive cells but also colocalizes with DC-LAMP itself, consistent with previous *in vitro* reports of its localization to endosomal/lysosomal membranes (Figure 5A). In contrast, ciliated respiratory epithelium shows intense labeling of the apical cytoplasm or plasma membrane rather than the punctate intracytoplasmic distribution typical of lysosomes (demonstrated by lysosomal marker MAC-3; Figure 5B). Whether Ifitm3 functions differently in the context of these cells or its apical localization is the end result of endo/lysosomal exocytosis is unknown.

While this manuscript was under review, Everitt et al. published data showing that *Ifitm3*-specific knockout mice are more susceptible to infection with influenza A virus X31 (H3N2) than wild type controls [26]. They also identified and characterized a polymorphism in human *IFITM3* associated with reduced restriction activity and found an increased prevalence of this mutant allele in patients hospitalized for influenza. Our data support and extend the core conclusions of their mouse studies. We further show that mice lacking entire *Ifitm* locus exhibit a nearly identical disease course to those lacking *Ifitm3* alone, and that their respective heterozygote littermates exhibit a phenotype intermediate between knockout and wild type mice. Morever, Ifitm3 is expressed on key IAV target cells in the lungs, and among murine Ifitms, is uniquely induced by type I and II interferons. Collectively these data make clear a primary role for Ifitm3 in the control of influenza A virus in mice.

We also show that many tissues express basal levels of Ifitm3 proteins, and that at least two gp130-family cytokines – OSM and IL-6 – can induce its expression, even in the absence of an interferon response. These results suggest greater complexity in the *in vivo* regulation of other well-characterized interferon-stimulated genes as well. We further noted Ifitm3 expression in cell populations that are not major IAV targets such as macrophages and endothelial cells. This implies that *Ifitm3* deletion will likely enhance susceptibility to infections by monocyte-tropic flaviviruses and macrophage- and endotheliotropic filoviruses.

Our results may also have implications for treatment of viral infections. The fact that Ifitm3 expression can be induced by gp130-mediated cytokines may allow for modulation of its expression in a more targeted, less pleiotropic way by agonizing or antagonizing cell type-specific gp130 heterodimeric receptors. More generally, our findings suggest that pharmacologic induction of human IFITM3 expression or emulation of IFITM3 activity could provide a broad-spectrum therapeutic approach to the treatment of a range of human pathogenic viruses.

## Materials and Methods

### Ethics statement

This study was carried out in strict accordance with the recommendations in the Guide for the Care and Use of Laboratory Animals of the National Institutes of Health. These studies were approved by the Institutional Animal Care and Use Committee of Harvard Medical School (Protocol Number 04743).

### Influenza A virus

Human influenza virus A/PR/8 (H1N1) propagated in chicken eggs and sucrose gradient purified from allantoic fluid was obtained from Charles River Laboratories, aliquoted, and frozen. Titer was determined by standard plaque assay on Madin-Darby canine kidney cells.

### Mice and inoculations

Heterozygous *IfitmDel* and *Ifitm3egfp* mice (background strain C57BL/6J-Tyrc-2J/J) were generously provided by Dr. David Adams (The Wellcome Trust Sanger Institute, Cambridge, United Kingdom). These animals were housed under specific pathogen-free conditions prior to infection. All mice were between 8 and 10 weeks old with the exception of three 12-week-old littermates (one of each genotype) used in Figure 1A. Mice were anesthetized by intraperitoneal injection of a cocktail of ketamine (2 mg per 25 g body weight) and xylazine (0.2 mg per 25 g body weight) in 200 µl of normal saline. Anesthetized mice were inoculated intranasally with influenza A virus diluted in 50 µl of phosphate-buffered saline divided evenly between both nostrils and administered over the course of one to two minutes. Animals were weighed once daily and, per institutional requirements, euthanized upon loss of 20% of initial body weight or when deemed moribund based on clinical signs. All procedures were performed with the approval of Harvard University's Institutional Animal Care and Use Committee.

### Viral load measurements

Lungs and spleens were harvested, rapidly frozen, and stored at −80°C prior to RNA isolation. Tissue was thawed in RNALater, transferred to 1 ml (lungs) or 0.5 ml (spleens) of RNAase-free water and mechanically homogenized with a QIAGEN TissueRuptor. RNA extraction was performed by means of the QIAGEN RNeasy Tissue Kit and QIAshredder columns (QIAGEN). Viral loads were determined by real-time TaqMan RT-PCR. Influenza A viral M2-specific primer and probe sequences were adapted for use with IAV PR/8/34 from protocols developed by the Centers for Disease Control [27]. Sequences were: 5′-GAC CAA TCC TGT CAC CTC TGA C-3′ (forward primer), 5′-AGG GCA TTT TGG ACA AAG CGT CTA-3′ (reverse primer), and 5′-TGC AGT CCT CGC TCA CTG GGC ACG-3′ (probe). Reporter and quencher dyes were FAM and IBFQ respectively with an additional internal Zen Quencher (Integrated DNA Technologies). Similarly labeled Gapdh primer and probe sequences were: 5′-GTG GAG TCA TAC TGG AAC ATG TAG-3′ (forward primer), 5′-AAT GGT GAA GGT CGG TGT G-3′ (reverse primer), and 5′-TGC AAA TGG CAG CCC TGG TG-3′ (probe) (Integrated DNA Technologies). 25 µl reactions containing 25 ng RNA, 50 pmol each primer, and 25 pmol probe were prepared using the Superscript III One-Step RT-PCR System with Platinum Taq DNA Polymerase and ROX (Invitrogen). Cycling conditions were: 50°C for 30 min (reverse transcription), 95°C for 2 min (denaturation), and 45 cycles of 95°C for 15 seconds (dentaturation) and 55°C for 45 sec (annealing, extension, and fluorescence acquisition). Cycling and fluorescence detection were performed in an ABI Prism 7500 thermal cycler (Applied Biosystems).

### Pseudovirus entry assays

Entry assays were performed on murine embryonic fibroblasts (MEFs) derived from *IfitmDel* knockout mice. Cells were transduced with retroviral puromycin-selectable vectors encoding the murine Ifitm proteins or with an empty vector control, as previously described [4]. MEFs were cultured for one week in puromycin to correct for variation in transduction efficiency. GFP-encoding retroviruses pseudotyped with PR8 neuraminidase and IAV HA (derived from influenza A/PR/8/34 (H1N1), influenza A/Thailand/2(SP-33)/2004 (H5N1), and A/FPV/Rostock (H7N1)) or the envelope glycoprotein of MLV or LASV were prepared as previously described [11], [28]. Ifitm-expressing MEFs were incubated with pseudoviral particles by spin-inoculation at 4000×g for 30 minutes at 4°C. Cells were washed and returned to growth medium. 48 hours later, cells were harvested, fixed in 1% paraformaldehyde, and GFP expression was quantified by flow cytometry.

### Western blots

RAW264.7 cells and 3T3 cells were incubated with varying concentrations of IFNα2 (eBioscience), IFNγ2 (Antigenix America), IFNγ (Antigenix America), IL-6 (Sigma), or OSM (Sigma). Two days later, cells were lysed with 1% NP40 and lysates were analyzed by SDS-PAGE and western blot. Goat anti-mouse Ifitm3 antibody (R & D systems) and HRP-conjugated rabbit anti-goat IgG secondary antibody (Sigma) were used to detect the expression of Ifitm3. Murine anti-β-tubulin antibody (Sigma) and HRP-conjugated rabbit anti-mouse IgG secondary antibody (Santa Cruz Biotechnology) were used to measure the expression of tubulin as a loading control. Expression of myc-tagged murine Ifitms was determined using the monoclonal 9E10 antibody (Santa Cruz Biotechnology).

### Flow cytometry

5×10^5^ NIH 3T3 or RAW 264.7 cells were stained with a panel of rat monoclonal IgG_2a_ antibodies against the OSM receptor (clone 30-1, MBL International Corporation), IL-6 receptor (clone 255821, R&D Systems), gp130 (clone 125623, R&D Systems), or an equal amount of an isotype control antibody (clone eBR2a, eBiosciences). Secondary labeling was performed with Alexa 488-conjugated donkey anti-rat (Invitrogen) and fluorescence was measured on a BD Biosceinces FACSCalibur flow cytometer.

### Promoter activity assays

Approximately 800 bp of each murine promoter region was amplified from wild type MEF genomic DNA and subcloned into the XhoI and HindIII sites of the luciferase reporter vector pGL3-Enhancer (Clontech). Primers used were as follows (promoter: forward/reverse): Ifitm1: 5′-CCC CAC ATA AAA GGT CAT GG-3′/5′-TCG GCT TTT GAA GCT GCA GA-3′, Ifitm2: 5′-CTC CTC CTT GCT CCA TTC TG-3′/5′-ACT GAC TCT GGA ACA ATC GC-3′, Ifitm3: 5′-GAG TGG CTG TAG CAC CAA CA-3′/5′-GCG GAG CAA AGG CAG CAC-3′, Ifitm5: 5′-CCT CTT TGC CTG CTG TCT TC-3′/5′-TTC CAG CGC CGT GTC TTC C-3′, Ifitm6: 5′-CGA TCC TGT TTT GCC ATC TT-3′/5′-TTT GTG CTT AAA GGA AGC AAG GAA-3′, Ifitm7 5′-ATT GAG ATG GGG TTT CAC CA-3′/5′-TTG GTT TTT GAG GCT GGA AGA G-3′. NIH 3T3 cells were cultured in DMEM supplemented with 10% calf serum (Colorado Serum Company), non-essential amino acids, and penicillin/streptomycin. 3T3 cells in 12 well plates were cotransfected with 0.5 µg of the promoter/pGL3 construct and 0.5 µg of a glucokinase promoter-driven constitutive β-galactosidase reporter (pGK-β-gal) as a transfection efficiency control. 3T3 cell transfection was carried out using the TransIT-3T3 Transfection Kit (Mirus). Transfection complexes were removed after 6 hours and growth medium alone or medium containing 100 ng/mL murine IFNα2 (eBioscience) or IFNγ (Antigenix America) was added to the cells. After 24 hours of stimulation, cells were lysed and enzymatic activities quantitated by means of the commercial Luciferase Assay System and β-Galactosidase Enzyme Activity Kit (Promega) in a Victor^3^V plate reader (PerkinElmer). Untransfected wells were used to determine background signal. Data are presented as the background subtracted luciferase readout for each sample normalized to the background-subtracted β-galactosidase readout for that sample.

### Ifitm and IFN transcript expression

NIH 3T3 and RAW 264.7 cells were grown in 12-well plates. Triplicate wells containing subconfluent cells were incubated in growth medium alone or treated for 24 hours with medium containing 500 ng/mL IFNα2, 100 ng/mL IFNγ2, 250 ng/mL OSM (3T3 cells), or 250 ng/mL IL-6 (RAW cells). RNA was isolated from cells by means of the RNeasy Mini Kit and QIAshredder columns (QIAGEN). 3T3 cells used as controls for Figure S3 were transfected with 1 5 µg/ml high molecular weight poly I:C complexed with LyoVec transfection reagent (Invivogen) for 24 hours before harvest. Primer and probe sequences for detection of murine Ifitm1, 2, and 3 transcripts follow. Ifitm1: 5′-ACC ACA ATC AAC ATG CCT GA-3′ (forward primer), 5′-CAC CAT CTT CCT GTC CCT AGA-3′ (reverse primer), and 5′-ACA CTC TTC ATG AAC TTC TGC TGC CTG-3′ (probe), Ifitm2: 5′-TTT TCT CTA CCA CCT CTG TGG T-3′ (forward primer), 5′-TGA ATC CAC TGT GGA CAG ATA G-3′ (reverse primer), and 5′-CGG TCC ACA TCT GCC CCG CC-3′ (probe), and Ifitm3: 5′-CTG AAC ATC AGC ACC TTG GT-3′ (forward primer), 5′-TTT TGG TGG TTA TCA AGT GCA CT-3′ (reverse primer), and 5′-TCC GGT CCT GAA GTG CTT CAC CCT-3′ (probe). Murine IFNβ1 was amplified with: 5′-AGA TTC ACT ACC AGT CCC AGA-3′ (forward primer), 5′-TGA AGA CCT GTC AGT TGA TGC-3′ (reverse primer), and 5′-AGG CAA CCT TTA AGC ATC AGA GGC G-3′ (probe). Murine IFNγ1 was amplified with: 5′-TCC ACA TCT ATG CCA CTT GAG-3′ (forward primer), 5′-CTG AGA CAA TGA ACG CTA CAC A-3′ (reverse primer), and 5′-TTC CTC ATG GCT GTT TCT GGC TGT-3′ (probe). Gapdh (see Viral load measurements above) was used as a housekeeping control. Primers were 5′ labeled with FAM and included 3′ IBFQ and internal Zen quencher dyes (Integrated DNA Technologies). Cycling conditions were: 50°C for 30 min (reverse transcription), 95°C for 2 min (denaturation), and 45 cycles of 95°C for 15 seconds (dentaturation) and 61.5°C for 40 sec (annealing, extension, and fluorescence acquisition). All reactions were performed in duplicate.

### Immunohistochemistry, immunofluorescence, and confocal microscopy

Mouse lungs were inflated with 10% neutral buffered formalin, fixed for 24 hours, and stored in phosphate-buffered saline prior to paraffin embedding. 5 µm sections were deparaffinized and subjected to heat-mediated epitope retrieval in low pH Antigen Unmasking Solution (Vector Labs). Sections were stained for Ifitm3 (rabbit, Abcam), influenza A virus H1N1 (goat, Abcam), Mac-3 (rat IgG2a clone M3/84, BD Pharmingen), and DC-LAMP (rat IgG2a clone 1010E1.01, Imgenex). Secondary fluorescent labeling was performed with Alexa Fluor 488, 543, or 633-conjugated secondary antisera of donkey origin (Invitrogen). *Ifitm*-tranduced MEFs grown on 8 well chamber slides were fixed and permeabilized by immersion in a 1∶1 mixture of methanol and acetone at −20°C for 10 minutes prior to staining. Cells were stained with primary anti-myc clone 9E11 (Santa Cruz Biotechnology) and Alexa Fluor 488-conjugated goat anti-mouse secondary antibody (Invitrogen). Slides were coverslipped with ProLong Gold Antifade mounting medium containing DAPI (Invitrogen). Imaging was performed on a Leica TCS SP5 laser-scanning confocal microscope through a 63× 1.4 NA oil-immersion objective.

Brightfield immunohistochemistry for Ifitm3 was carried out with similar slide preparation and primary antibodies. Secondary labeling and detection were performed with biotinylated horse anti-rabbit, avidin/biotin/peroxidase complex, and Nova Red chromagen (Vector Labs). Digital images were color balanced for publication.

## Supporting Information

Figure S1 **Weight loss curves for mice in** **Figure 1** **.** Weight loss of knockout animals was more rapid and more uniform than that of heterozygous and wild type controls. (**A**) Weight loss over time is shown for individual animals in Figure 1A. Animals were euthanized at 20% weight loss (dotted line). (**B**) Panels show mean+SD of surviving animals from Figure 1A at each time point. The upward trend in wild type weights at day 8–9 reflects surviving and recovering animals. (**C**) Weight loss trends of animals from Figure 1C are plotted as above. Animals were euthanized on day 3 post-infection (dotted line) in order to measure viral loads. Heterozygotes in this cohort behaved similarly to wild type animals as reflected by both weight and viral load measurements. (**D**) Pulmonary and splenic viral loads from Figure 1C are plotted against a common log-scale axis to illustrate the magnitude of the differences between viral loads in these two organs. Values are shown relative to the lowest splenic viral load of the wild type mice.
(EPS)

Figure S2 **Ifitm3 expression and anti-IAV restriction activity are more efficient than other murine Ifitm proteins.** (**A**) *Ifitm*-transduced MEFs were challenged with pseudoviruses bearing various IAV HAs or control envelope glycoproteins from non-restricted viruses (MLV, LASV). The graph depicts pseudovirus infection of MEFs transduced to express the indicated myc-tagged Ifitm protein, normalized to the infection observed with MEFs transduced with vector alone. The percentage of infected vector-transduced cells for each pseudovirus is indicated below. Note that the IAV H1 shown here is derived from PR8, the virus used for the *in vivo* challenges in Figure 1. Bars indicate mean ± range of duplicates. (**B**) Expression levels of the myc-tagged Ifitm proteins in MEFs from panel (A) were determined by western blot using the anti-myc antibody 9E10. Ifitm3 is the most abundantly expressed. (**C**) *Ifitm*-transduced MEFs were also examined by confocal microscopy by staining the N-terminal myc tags (green). Nuclei were stained with DAPI (blue). Photomultiplier tube gain was adjusted individually for each panel in order to visualize the more poorly expressed proteins. All murine Ifitms display similar punctate intracytoplasmic staining. The non-specific green puncta visible within the nuclei of untransduced cells may represent staining of endogenous c-myc.
(EPS)

Figure S3 **OSM and IL-6 did not detectably upregulate expression of IFNβ1 or IFNγ.** RNA samples from untreated and OSM-treated NIH 3T3 cells and untreated and IL-6 treated RAW cells shown in Figure 3D were assayed for IFNβ1 and IFNγ expression by qRT-PCR. Untreated and poly I:C-treated 3T3 cells were included as a positive control for IFN stimulation. (**A**) 24 hours of OSM stimulation did not upregulate IFNβ1 mRNA in 3T3 cells. IL-6 induced a slight but non-significant increase in IFNβ1 transcript levels. Values are normalized to those of untreated cells. (**B**) IFNγ expression was below the threshold of detection in both 3T3 and RAW cells regardless of cytokine treatment. Only poly I:C stimulation resulted in detectable levels of IFNγ transcript, and all values are normalized to this level. Bars show mean ± range of triplicates.
(EPS)

Figure S4 **The antibody used in** **Figures 4** **and** **5** **is specific for Ifitm3.** Immunohistochemical staining for Ifitm3 was performed on lung tissue from an *Ifitm3* heterozygous mouse (top panels) and an *Ifitm3*-specific knockout littermate (bottom panels) infected with 500 PFU of PR8 and euthanized 3 DPI. Lack of staining of the *Ifitm3*-specific knockout mouse confirms the specificity of the antibody used in Figure 4 for Ifitm3. (**A**, **B**) Ifitm3 is localized to the apical cytoplasm or plasma membrane of bronchiolar epithelial cells as well as luminal inflammatory cells. (**C**, **D**) Lower airway epithelial cells express Ifitm3. (**E**, **F**) The visceral pleura is strongly Ifitm3 positive.
(TIF)

## References

[1] Schoggins JW, Wilson SJ, Panis M, Murphy MY, Jones CT, et al (2011) A diverse range of gene products are effectors of the type I interferon antiviral response. Nature 472: 481–485. 21478870 10.1038/nature09907 PMC3409588

[2] Brass AL, Huang IC, Benita Y, John SP, Krishnan MN, et al (2009) The IFITM proteins mediate cellular resistance to influenza A H1N1 virus, West Nile virus, and dengue virus. Cell 139: 1243–1254. 20064371 10.1016/j.cell.2009.12.017 PMC2824905

[3] Shapira SD, Gat-Viks I, Shum BOV, Dricot A, de Grace MM, et al (2009) A physical and regulatory map of host-influenza interactions reveals pathways in H1N1 infection. Cell 139: 1255–1267. 20064372 10.1016/j.cell.2009.12.018 PMC2892837

[4] Huang IC, Bailey CC, Weyer JL, Radoshitzky SR, Becker MM, et al (2011) Distinct patterns of IFITM-mediated restriction of filoviruses, SARS coronavirus, and influenza A virus. PLoS Pathog 7: e1001258–e1001258. 21253575 10.1371/journal.ppat.1001258 PMC3017121

[5] Jiang D, Weidner JM, Qing M, Pan XB, Guo H, et al (2010) Identification of five interferon-induced cellular proteins that inhibit west nile virus and dengue virus infections. J Virol 84: 8332–8341. 20534863 10.1128/JVI.02199-09 PMC2916517

[6] Weidner JM, Jiang D, Pan X-B, Chang J, Block TM, et al (2010) Interferon-induced cell membrane proteins, IFITM3 and tetherin, inhibit vesicular stomatitis virus infection via distinct mechanisms. J Virol 84: 12646–12657. 20943977 10.1128/JVI.01328-10 PMC3004348

[7] Alber D, Staeheli P (1996) Partial inhibition of vesicular stomatitis virus by the interferon-induced human 9–27 protein. J Interferon Cytokine Res 16: 375–380. 8727077 10.1089/jir.1996.16.375

[8] Lu J, Pan Q, Rong L, He W, Liu S-L, et al (2011) The IFITM proteins inhibit HIV-1 infection. J Virol 85: 2126–2137. 21177806 10.1128/JVI.01531-10 PMC3067758

[9] Feeley EM, Sims JS, John SP, Chin CR, Pertel T, et al (2011) IFITM3 inhibits influenza A virus infection by preventing cytosolic entry. PLoS Pathog 7: e1002337. 22046135 10.1371/journal.ppat.1002337 PMC3203188

[10] Wee YS, Roundy KM, Weis JJ, Weis JH (2012) Interferon-inducible transmembrane proteins of the innate immune response act as membrane organizers by influencing clathrin and v-ATPase localization and function. Innate Immun E-pub ahead of print. 10.1177/1753425912443392 22467717

[11] Huang IC, Bosch BJ, Li F, Li W, Lee KH, et al (2006) SARS coronavirus, but not human coronavirus NL63, utilizes cathepsin L to infect ACE2-expressing cells. J Biol Chem 281: 3198–3203. 16339146 10.1074/jbc.M508381200 PMC8010168

[12] Hickford DE, Frankenberg SR, Shaw G, Renfree MB (2012) Evolution of vertebrate interferon inducible transmembrane proteins. BMC Genomics 13: 155. 22537233 10.1186/1471-2164-13-155 PMC3424830

[13] Lange UC, Adams DJ, Lee C, Barton S, Schneider R, et al (2008) Normal Germ Line Establishment in Mice Carrying a Deletion of the Ifitm/Fragilis Gene Family Cluster. Mol Cell Biol 28: 4688–4696. 18505827 10.1128/MCB.00272-08 PMC2493357

[14] Moffatt P, Gaumond M-H, Salois P, Sellin K, Bessette M-C, et al (2008) Bril: A Novel Bone-Specific Modulator of Mineralization. J Bone Miner Res 23: 1497–1508. 18442316 10.1359/jbmr.080412

[15] Hanagata N, Li X, Morita H, Takemura T, Li J, et al (2011) Characterization of the osteoblast-specific transmembrane protein IFITM5 and analysis of IFITM5-deficient mice. J Bone Miner Metab 29: 279–290. 20838829 10.1007/s00774-010-0221-0

[16] Price GE, Gaszewska-Mastarlarz A, Moskophidis D (2000) The role of alpha/beta and gamma interferons in development of immunity to influenza A virus in mice. J Virol 74: 3996–4003. 10756011 10.1128/jvi.74.9.3996-4003.2000 PMC111913

[17] Klenk HD, Garten W (1994) Host cell proteases controlling virus pathogenicity. Trends Microbiol 2: 39–43. 8162439 10.1016/0966-842x(94)90123-6

[18] García-Sastre A, Durbin RK, Zheng H, Palese P, Gertner R, et al (1998) The role of interferon in influenza virus tissue tropism. J Virol 72: 8550–8558. 9765393 10.1128/jvi.72.11.8550-8558.1998 PMC110265

[19] Sun X, Tse LV, Ferguson AD, Whittaker GR (2010) Modifications to the hemagglutinin cleavage site control the virulence of a neurotropic H1N1 influenza virus. J Virol 84: 8683–8690. 20554779 10.1128/JVI.00797-10 PMC2919019

[20] Bottcher E, Matrosovich T, Beyerle M, Klenk HD, Garten W, et al (2006) Proteolytic activation of influenza viruses by serine proteases TMPRSS2 and HAT from human airway epithelium. J Virol 80: 9896–9898. 16973594 10.1128/JVI.01118-06 PMC1617224

[21] Larrea E, Aldabe R, Gonzalez I, Segura V, Sarobe P, et al (2009) Oncostatin M enhances the antiviral effects of type I interferon and activates immunostimulatory functions in liver epithelial cells. J Virol 83: 3298–3311. 19158240 10.1128/JVI.02167-08 PMC2655580

[22] Silver JS, Hunter CA (2010) gp130 at the nexus of inflammation, autoimmunity, and cancer. J Leukoc Biol 88: 1145–1156. 20610800 10.1189/jlb.0410217 PMC2996896

[23] Friedman RL, Manly SP, McMahon M, Kerr IM, Stark GR (1984) Transcriptional and posttranscriptional regulation of interferon-induced gene expression in human cells. Cell 38: 745–755. 6548414 10.1016/0092-8674(84)90270-8

[24] Ibricevic A, Pekosz A, Walter MJ, Newby C, Battaile JT, et al (2006) Influenza virus receptor specificity and cell tropism in mouse and human airway epithelial cells. J Virol 80: 7469–7480. 16840327 10.1128/JVI.02677-05 PMC1563738

[25] Salaun B, de Saint-Vis B, Pacheco N, Pacheco Y, Riesler A, et al (2004) CD208/dendritic cell-lysosomal associated membrane protein is a marker of normal and transformed type II pneumocytes. Am J Pathol 164: 861–871. 14982840 10.1016/S0002-9440(10)63174-4 PMC1613301

[26] Everitt AR, Clare S, Pertel T, John SP, Wash RS, et al (2012) IFITM3 restricts the morbidity and mortality associated with influenza. Nature 484: 519–523. 22446628 10.1038/nature10921 PMC3648786

[27] World Health Organization (2009) CDC protocol of realtime RTPCR for influenza A (H1N1). Geneva: World Health Organization. Available: http://www.who.int/csr/resources/publications/swineflu/realtimeptpcr/en/index.html.

[28] Huang IC, Li W, Sui J, Marasco W, Choe H, et al (2008) Influenza A virus neuraminidase limits viral superinfection. J Virol 82: 4834–4843. 18321971 10.1128/JVI.00079-08 PMC2346733

