# Supplementary figures and images for: Ifitm3 Limits the Severity of Acute Influenza in Mice
Source: PLoS Pathog. 2012 Sep 6;8(9):e1002909. doi: 10.1371/journal.ppat.1002909 (PMC3435252; doi:10.1371/journal.ppat.1002909)

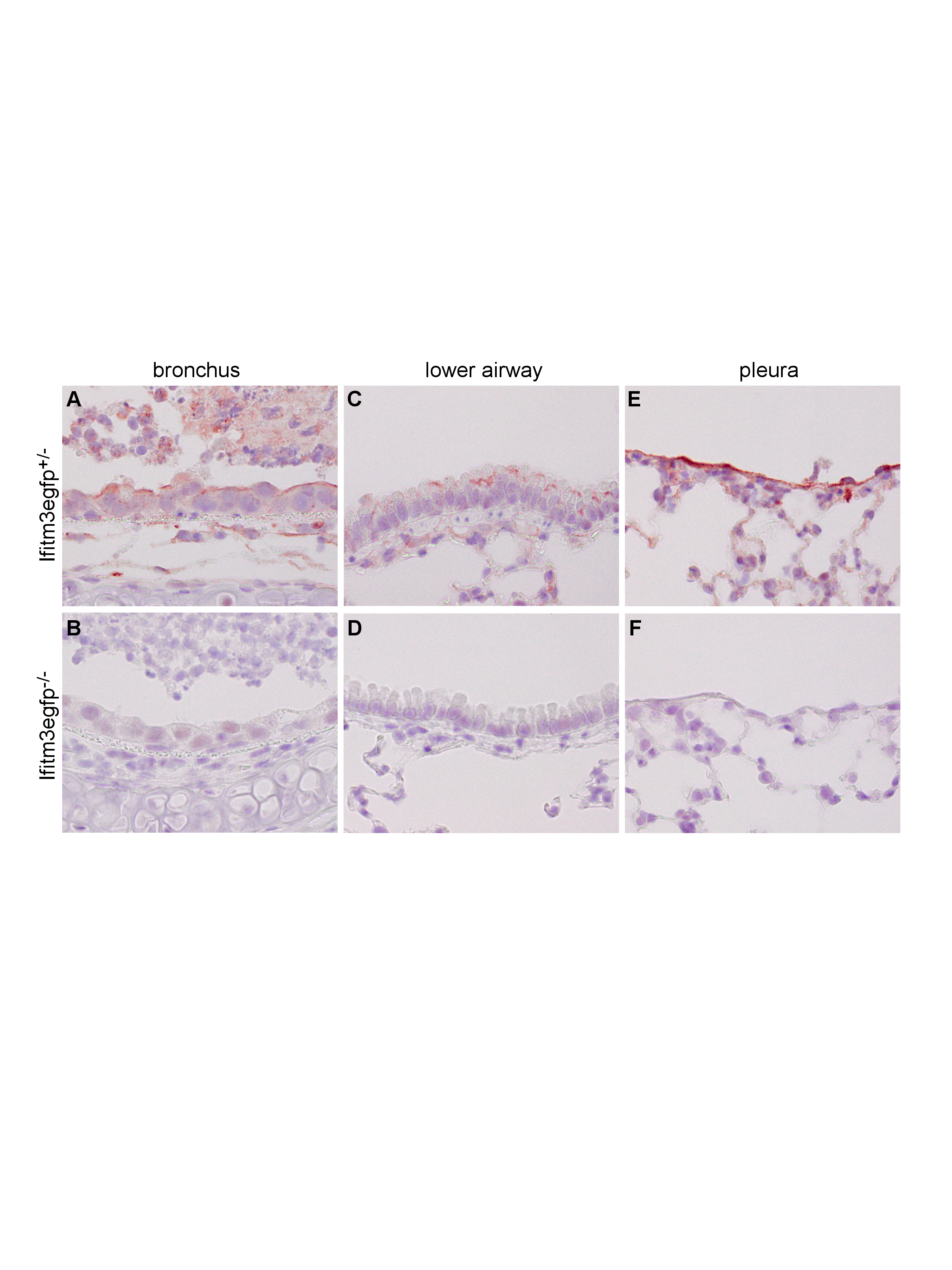

Supplement: Figure S4 — The antibody used in Figures 4 and 5 is specific for Ifitm3. Immunohistochemical staining for Ifitm3 was performed on lung tissue from an Ifitm3 heterozygous mouse (top panels) and an Ifitm3-specific knockout littermate (bottom panels) infected with 500 PFU of PR8 and euthanized 3 DPI. Lack of staining of the Ifitm3-specific knockout mouse confirms the specificity of the antibody used in Figure 4 for Ifitm3. (A, B) Ifitm3 is localized to the apical cytoplasm or plasma membrane of bronchiolar epithelial cells as well as luminal inflammatory cells. (C, D) Lower airway epithelial cells express Ifitm3. (E, F) The visceral pleura is strongly Ifitm3 positive. (TIF) [file ppat.1002909.s004.tif]
